# Long-term trajectories of invasive hemodynamic parameters and clinical outcomes after durable left ventricular assist device implantation

**DOI:** 10.1016/j.jhlto.2026.100711

**Published:** 2026-10-21

**Authors:** Stijn Legtenberg, Jozine M. ter Maaten, Michiel E. Erasmus, Michiel Kuijpers, Yvonne L. Douglas, Joep M. Droogh, Eva de Felice, Stan A.J. van den Broek, Jan A. Krikken, Adriaan A. Voors, Kevin Damman

**Affiliations:** a University of Groningen, Department of Cardiology, University Medical Center Groningen, Groningen, the Netherlands; b University of Groningen, Department of Cardiothoracic Surgery, University Medical Center Groningen, Groningen, the Netherlands; c University of Groningen, Department of Critical Care, University Medical Center Groningen, Groningen, the Netherlands

**Keywords:** ventricular assist devices, long-term hemodynamics, right heart catheterization, advanced heart failure, right ventricular dysfunction

## Abstract

**Background:**

Long-term hemodynamic changes after durable left ventricular assist device (LVAD) implantation are not well characterized. Characterizing these trajectories is clinically relevant, as persistent or evolving abnormalities in filling pressures and pulmonary circulation may identify patients at risk for adverse outcomes.

**Method:**

We conducted a retrospective single-center study including all patients who underwent durable LVAD implantation at the University Medical Center Groningen (2012 to 2025). We evaluated serial right heart catheterization (RHC) measurements to describe hemodynamic trajectories. Repeated measures, mixed-effect models were used to analyze hemodynamic trajectories up to 60 months of follow-up, including mean pulmonary artery pressure (mPAP), pulmonary capillary wedge pressure (PCWP), right atrial pressure (RAP), and pulmonary artery pulsatility index (PAPi). Trajectories were compared according to survivor status, baseline LVAD strategy, development of late right ventricular failure (RVF), presence of aortic regurgitation (AR), and worsening renal function (WRF).

**Results:**

Among 199 patients (176 HeartMate 3; 23 HeartMate II), mean PAP (−10.1 mmHg, 95% CI −13.1 to −7.1) and PCWP (−9.9 mmHg, 95% CI −12.5 to −7.2) decreased significantly within the first 6 months after LVAD implantation, while RAP (0.0 mmHg, 95% CI −0.8 to 0.8) remained unchanged. Cardiac index (+0.6 L/min/m², 95% CI 0.4−0.8) and cardiac output (+1.3 L/min, 95% CI 0.9−1.7) increased significantly over the same interval. Beyond 6 months, trajectories for most hemodynamic parameters remained generally stable. Non-survivors and patients developing late RVF showed consistently higher filling pressures with trajectories largely parallel to those of survivors or those without RVF. Hemodynamic trajectories did not differ by initial LVAD strategy. In contrast, clinically significant aortic regurgitation and WRF were associated with distinct hemodynamic trajectories.

**Conclusion:**

Hemodynamic parameters after durable LVAD implantation improved early and remained largely stable thereafter. Patients with adverse clinical outcomes showed persistently higher and/or altered hemodynamic profiles. These findings suggest that longitudinal hemodynamic assessment may enable early identification of patients at risk for adverse outcomes.

An estimated 1% to 10% of patients with heart failure with reduced ejection fraction (HFrEF) can be classified as having advanced heart failure.^1^ Durable left ventricular assist device (LVAD) support has become a cornerstone treatment in the management of advanced heart failure, offering improved survival and quality of life in patients who are no longer adequately treated by medical therapy alone.^2^, ^3^, ^4^ The current generation LVAD, the HeartMate 3, has further advanced outcomes due to enhanced durability and markedly reduced rates of hemocompatibility-related adverse events.^5^, ^6^, ^7^ While its impact on end-organ perfusion and functional capacity has been well demonstrated, our understanding of long-term hemodynamic behavior after durable LVAD implantation remains incomplete.

Hemodynamic unloading of the left ventricle is an immediate and expected response to LVAD support, reflected by early reductions in filling pressures and pulmonary artery pressures in the postoperative phase.^8^, ^9^, ^10^ However, much less is known about how these parameters evolve during prolonged support. Particularly in countries like the Netherlands, the considerable shortage of donor hearts can lead to long waiting times for heart transplantation, thereby prolonging the need for mechanical circulatory support (MCS), especially in tall individuals with common blood type.^11^ In parallel, the destination-therapy population is steadily expanding, and improved device durability has enabled long-term survival beyond what was previously achievable.^12^, ^13^ These developments emphasize the need to better understand long-term hemodynamic changes during continuous-flow LVAD therapy.

Understanding hemodynamic trajectories of filling pressures during LVAD support is clinically important, as optimal hemodynamics have been linked to better outcomes, such as reduced hemocompatibility-related adverse events and lower readmission rates.^14^, ^15^, ^16^ Right ventricular adaptation and effective reduction of pulmonary afterload appear to be key determinants of stable long-term LVAD physiology, as the right ventricle becomes highly sensitive to afterload in this setting, such that even modest elevations can impair forward flow.^17^

In this study, we present novel insights into long-term hemodynamic trajectories during LVAD support by analyzing serial right-heart catheterization (RHC) measurements before and throughout chronic LVAD therapy, predominantly in patients supported with the HeartMate 3 device. Our aim was to characterize longitudinal changes in cardiac pressures and right ventricular-related hemodynamic indices, and to determine how these trajectories are associated with clinical outcomes, including survival, right ventricular failure (RVF), worsening renal function (WRF), and aortic regurgitation (AR).

## Methods

We included all patients who were implanted with a Heartmate II (HMII) or HeartMate 3 (HM3) LVAD at the University Medical Center Groningen, from the first implantation on November 26, 2012, to data collection ending on March 1, 2025. In the Netherlands, inclusion of all patients in the European Registry for Patients with Mechanical Circulatory Support (EUROMACS) is mandatory following the acquisition of signed informed consent.^18^, ^19^ Patients were included regardless of any concomitant procedures performed at the time of implantation. All patients provided informed consent for inclusion in EUROMACS and associated research as described above; data for the present study were collected from institutional clinical records and databases. All patients were evaluated and managed according to the local institutional standard of care.

### Objectives

The primary objective was to characterize longitudinal changes in invasive hemodynamic parameters during continuous-flow LVAD support, using serial RHC measurements up to 60 months post-implant, as later time points contained limited observations. Secondary objectives examined differences in hemodynamic trajectories according to survival status, initial LVAD strategy (bridge to transplant, destination therapy, or bridge to decision), the occurrence of late RVF, presence of clinically significant AR, and WRF. Exploratively, longitudinal changes in NYHA functional class were assessed to provide complementary information on functional status during follow-up.

### Definitions

Late RVF was defined according to MCS-ARC criteria as any of the following occurring more than 30 days after LVAD implantation: need for right ventricular assist device (RVAD) support, hospital admission requiring intravenous diuretics or inotropic therapy ≥72 hours, with accompanying clinical signs of right-heart failure.^20^ Clinically significant AR was defined as a regurgitation severity of moderate or severe during follow-up as assessed by echocardiography or the need for aortic valve replacement.^21^ WRF was defined as a >30% decrease in estimated glomerular filtration rate (eGFR) during follow-up compared with the post-LVAD implantation discharge value, in line with prior studies defining WRF as a 20% to 30% decline in eGFR or equivalent changes in creatinine.^22^ eGFR was calculated using the CKD-EPI 2021 creatinine equation^23^, based on serum creatinine measurements obtained during routinely scheduled outpatient follow-up visits.

### Right heart catheterization

RHC was performed according to institutional clinical practice. In patients listed for heart transplantation RHC was routinely repeated at regular intervals (typically every 6-12 months) during follow-up. In patients classified as bridge-to-decision (BTD), repeat RHC was routinely performed to support reclassification to either bridge-to-transplant (BTT) or destination therapy (DT). In patients receiving LVAD as DT, repeat RHC was performed only when clinically indicated and was not protocol-mandated. Consequently, patients with clinical deterioration or suspected device-related complications may have been overrepresented within the DT population. Therefore, serial hemodynamic measurements should primarily be interpreted as descriptive of the studied population rather than representative of all LVAD recipients.

Hemodynamic parameters included mean, systolic and diastolic pulmonary artery pressure (mPAP, sPAP, dPAP), pulmonary capillary wedge pressure (PCWP), right atrial pressure (RAP), systemic and pulmonary vascular resistance (SVR, PVR), cardiac output (CO), cardiac index (CI), right-ventricular stroke-work index (RVSWI) and pulmonary artery pulsatility index (PAPi). If RAP was recorded as 0 mmHg (0.7% of RHCs), this was replaced by 1 mmHg to allow calculation of PAPi only, in order to avoid an infinitely high PAPi.

### Statistical analysis

Normally distributed continuous variables are presented as mean ± standard deviation, and non-normally distributed variables as median with interquartile range (IQR; 25th to 75th percentile). Categorical variables are summarized as counts and percentages [*n (%)*].

For descriptive analyses, serial hemodynamic measurements were categorized according to the chronological sequence of RHCs (i.e., first post-LVAD RHC, second post-LVAD RHC, etc.), irrespective of the exact timing after implantation. Consequently, the timing of RHCs varied between patients, and the median time from LVAD implantation to each sequential RHC is reported. NYHA functional class was summarized descriptively among patients with available assessments at each follow-up time point.

For each parameter, longitudinal trajectories were modeled using repeated measures, linear mixed-effects models with patient-specific random intercepts. All available RHC measurements were included in the mixed-effects models, thereby accounting for irregular follow-up intervals and unequal numbers of observations per patient. No imputation of missing hemodynamic measurements was performed. Models including random slopes were compared with random-intercept-only models using likelihood ratio testing and retained when model fit improved significantly. Non-linearity was evaluated by comparing linear, quadratic, and spline model specifications. Restricted cubic splines were applied using the optimal number of degrees of freedom determined by the lowest Akaike Information Criterion (AIC). The final model per parameter was therefore either linear, quadratic, or spline-based, depending on the best fit. The same modeling approach was applied in all subgroup analyses.

Estimated marginal means with 95% confidence intervals were generated at predefined time points, including baseline (time 0) and postoperative follow-up at 6, 12, 24, 36, 48, and 60 months. Baseline values were derived from the pre-LVAD RHC; if the pre-LVAD catheterization was unavailable, the date of LVAD implantation was used as the baseline time point. Differences between clinically relevant time points were evaluated using pre-specified contrasts of estimated marginal means derived from the mixed-effects models. Specifically, contrasts were defined a priori to assess early hemodynamic response (baseline vs. 6 months) and long-term stability (6 vs 60 months). As these contrasts were hypothesis-driven and not exploratory pairwise comparisons across all time points, no adjustment for multiple testing was applied. Model improvement and interaction effects were assessed using likelihood ratio tests (LRT). *p*-values <0.10 were considered evidence of interaction, and interaction terms meeting this criterion were retained in the model. Predicted population trajectories with corresponding 95% confidence intervals were visualized by plotting estimated marginal means across time.

Two tailed *p*-values < 0.05 were considered statistically significant. All statistical analyses were performed using R version 4.5.0 (R Foundation for Statistical Computing, Vienna, Austria).

## Results

### Baseline patient characteristics

The study population consisted of a total of 199 patients who were implanted with either a HMII (N=23) or HM3 (N=176) during the study period. The mean age at implantation was 55 ± 11 years, and 25% of patients were female. Baseline characteristics are summarized in Table 1.

**Table 1:** Baseline Characteristics of the Study Population Before LVAD Implantation

	**UMCG LVAD cohort**
	***N=199***
LVAD type (n(%))	
*HeartMate II*	23 (12)
*HeartMate 3*	176 (88)
LVAD strategy (n(%))	
*Bridge to Transplant*	58 (29)
*Bridge to Decision*	74 (37)
*Destination therapy*	67 (34)
Concomitant Surgery (n(%))	
*Congenital (ASD/VSD/PFO closure)*	7 (4)
*Valve surgery (bio-AVR, aortic repair)*	33 (18)
*Other (CABG, ECMO, unspecified)*	11 (6)
Age (years)	55.4 (11.1)
Female (n(%))	50 (25)
Ethnicity (Caucasian n (%))	192 (97)
BMI (kg/m^2^)	26.1 (4.7)
Etiology of heart failure (n(%))	
*Congenital Heart Disease*	2 (1)
*Dilated Cardiomyopathy*	117 (59)
*Hypertrophic Cardiomyopathy*	3 (2)
*Ischemic*	70 (35)
*Restrictive Cardiomyopathy*	4 (2)
*Valvular*	3 (2)
INTERMACS level (n(%)):	
*2 - Progressive Decline*	42 (21)
*3 - Stable but inotrope dependent*	102 (51)
*4 - Resting Symptoms*	47 (24)
*5 - Exertion Intolerant*	8 (4)
Time since first HF diagnosis (n(%))	
*<1 month*	35 (18)
*1 month - 1 year*	23 (12)
*1-2 years*	12 (6)
*> 2 years*	129 (65)
**Echocardiography**	
TAPSE (mm)	16.2 (4.1)
Mitral regurgitation (n(%)):	
*Mild or less*	79 (40)
*Moderate-Severe*	118 (60)
Tricuspid regurgitation (n(%))	
*Mild or less*	123 (63)
*Moderate-Severe*	71 (37)
Aortic regurgitation (n(%))	
*Mild or less*	192 (98)
*Moderate-Severe*	3 (2)
**Medical History**	
Diabetes (n(%))	37 (19)
COPD (n(%))	14 (7)
CVA/TIA (n(%))	16 (8)
Cancer other than local skin cancer (n(%))	14 (7)
Atrial fibrillation (n(%))	64 (32)
Ventricular arrhythmias (n(%))	35 (18)
Previous heart surgery	49 (25)
**Medical therapy**	
Inotropes before implant (Milrinone, n(%))	188 (96)
ARB (n(%))	36 (18)
ACEi (n(%))	45 (23)
Beta-blocker (n(%))	118 (60)
MRA (n(%))	161 (81)
Loop Diuretics (n(%))	179 (90)
Amiodarone (n(%))	78 (39)
ICD (n(%))	128 (64)
Temporary MCS pre-LVAD (n(%))	18 (9)
**Laboratory**	
NT-proBNP (pg/mL)	4858 [2679;7883]
Hemoglobin (mmol/L)	7.6 (1.4)
Sodium (mmol/L)	137 (4.6)
Creatinine (µmol/L)	1.34 [1.06;1.63]
eGFR (mL/min/1.73 m2)	63.4 (25.8)
Length of stay after implantation (days)	24.0 [18.0;33.0]

BMI, body mass index; BTD, bridge to decision; BTT, bridge to transplant; DT, destination therapy; HM III, heartmate 3; COPD, chronic obstructive pulmonary disease; CVA, cerebrovascular accident; TIA, transient ischemic attack; ICD, implantable cardioverter defibrillator; INTERMACS, Interagency Registry for Mechanically Assisted Circulatory Support; LVAD, left ventricular assist device; LVEF, left ventricular ejection fraction; ACEi, angiotensin-converting enzyme inhibitors; ARB, angiotensin II receptor blocker; LOS, Length of Stay; MRA, mineralocorticoid receptor antagonist; NYHA, New York Heart Association class of heart failure; TAPSE, tricuspid annular plane systolic excursion; eGFR, estimated Glomerular Filtration Rate; MCS, mechanical circulatory support; HF, heart failure; ASD, atrial septal defect; PFO, patent foramen ovale; ECMO, extracorporeal membrane oxygenation; CABG, coronary artery bypass grafting

The number of RHCs per time point is shown in Table 2. The total number of RHCs for the entire study was 537 (median per patient 2 1, 2, 3, 4). Supplementary Figure 1 provides an overview of RHC availability per time point, stratified by initial LVAD strategy, demonstrating that long-term RHC follow-up was observed almost exclusively in patients implanted as BTT as local protocol mandated. Accordingly, patients receiving LVADs as DT had the shortest duration of RHC follow-up.

**Table 2:** Availability and Timing of Sequential Right Heart Catheterizations

**RHC timepoint**	**N**	**Median interval (months) between RHCs**
Pre-LVAD	187	
		8.5 [5.9-11.9]
First post-LVAD	123	
		7.1 [6.2-9.3]
Second post-LVAD (median 1.3y)	79	
		6.9 [6.0-9.6]
Third post-LVAD (median 1.8y)	56	
		6.7 [6.1-8.0]
Fourth post-LVAD (median 2.4y)	39	
		7.1 [6.4-9.3]
Fifth post-LVAD (median 3y)	23	
		7.7 [6.2-10.1]
Sixth post-LVAD (median 3.6y)	16	
		9.6 [7.8-12.5]
Seventh post-LVAD (median 4.4y)	9	
		8.8 [6.6-11.3]
Eighth post-LVAD (median 5.1y)	5	

RHCs are presented according to the chronological sequence in which they occurred (before and) after LVAD implantation, irrespective of the exact timing after implantation. The number of RHCs therefore reflects the availability of invasive hemodynamic measurements rather than the number of patients remaining under follow-up

Median time from RHC to LVAD-implant: 16 [9-52] days

Median time from LVAD-implant to RHC: 7 [5-10] months

Median total FU RHC: 9 [0-27] months

Max FU RHC: 9y (110 months)

RHC timepoints > 9 were excluded due to insufficient sample size (n=2)

During follow-up, 66 patients died, 48 developed late RVF, 19 developed clinically significant AR, and 74 experienced WRF. The number of events and median time to event for each outcome are summarized in Supplementary Table 1.

### Hemodynamic trajectories over time after LVAD implantation

LVAD implantation was associated with pronounced early hemodynamic changes, followed by long-term stabilization (Figure 1 A-G, Table 3). Pulmonary pressures and left sided filling pressures decreased significantly within 6 months, including mPAP (−10.1 mmHg, 95% CI −7.1 to −13.1), sPAP (−12.9 mmHg, 95% CI −9.8 to −16.0), dPAP (−8.5 mmHg, 95% CI −5.9 to −11.1), and PCWP (−9.9 mmHg, 95% CI −7.2 to −12.5; all *p*<0.0001). Beyond 6 months, pulmonary pressures remained stable, whereas PCWP showed a modest increase between 6 and 60 months (+2.9 mmHg, 95% CI 0.6-5.3; *p*<0.013). Overall, mean RAP remained unchanged throughout follow-up.

**Figure 1:**
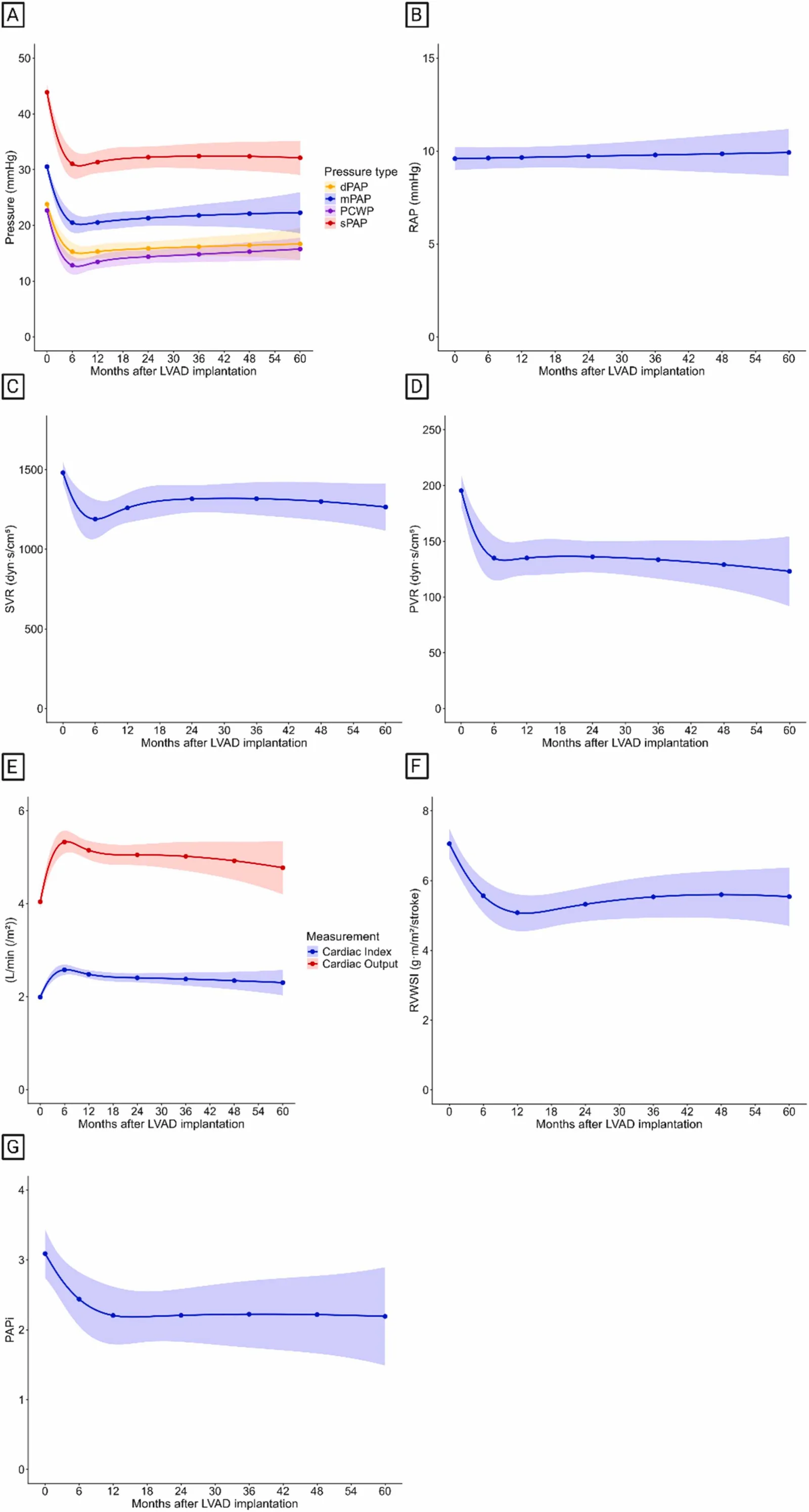
Right-sided hemodynamic trajectories in full cohort. A – Pulmonary artery pressures and PCWP; B – RAP; C – SVR; D – PVR; E – CO/CI; F – RVSWI; G – PAPi. Trajectories represent model-estimated marginal means derived from linear mixed-effects models using all available RHC measurements at their actual follow-up times. Displayed time points do not represent discrete study visits or measurements from a fixed patient cohort. CO, cardiac output; CI, cardiac index; dPAP, diastolic pulmonary artery pressure; LVAD, left ventricular assist device; mPAP, mean pulmonary artery pressure; PAPi, pulmonary artery pulsatility index; PCWP, pulmonary capillary wedge pressure; PVR, pulmonary vascular resistance; RAP, right atrial pressure; RVSWI, right ventricular stroke work index; sPAP, systolic pulmonary artery pressure; SVR, systemic vascular resistance

**Table 3:** Pairwise Comparisons of Predicted Hemodynamic Values Over Time

**Parameter**	**Timepoint**	**Compared to**	**Change (95% CI)**	**P-value**
mPAP (mmHg)	Pre-LVAD	6 months	−10.1 (−7.1 to −13.1)	<0.0001
	6 months	60 months	1.7 (−2.2 to 5.6)	0.38
sPAP (mmHg)	Pre-LVAD	6 months	−12.9 (−9.8 to −16.0)	<0.0001
	6 months	60 months	1.1 (−2.3 to 4.6)	0.53
dPAP (mmHg)	Pre-LVAD	6 months	−8.5 (−5.9 to −11.1)	<0.0001
	6 months	60 months	1.3 (−1.6 to 4.2)	0.38
PCWP (mmHg)	Pre-LVAD	6 months	−9.9 (−7.2 to −12.5)	<0.0001
	6 months	60 months	2.9 (0.6 to 5.3)	0.013
RAP (mmHg)	Pre-LVAD	6 months	0.0 (−0.1 to 0.2)	0.68
	6 months	60 months	0.3 (−1.0 to 1.5)	0.68
CO (L/min)	Pre-LVAD	6 months	1.3 (1.0 to 1.6)	<0.0001
	6 months	60 months	−0.6 (−1.2 to 0.0)	0.06
CI (L/min/m²)	Pre-LVAD	6 months	0.6 (0.5 to 0.7)	<0.0001
	6 months	60 months	−0.3 (−0.6 to 0.0)	0.06
SVR (dyn·s/cm)	Pre-LVAD	6 months	−292 (−422 to −161)	<0.0001
	6 months	60 months	76 (−102 to 254)	0.40
PVR (dyn·s/cm)	Pre-LVAD	6 months	−62 (−82 to −42)	<0.0001
	6 months	60 months	−13 (−47 to 22)	0.46
RVSWI (g·m/m²/stroke)	Pre-LVAD	6 months	−1.5 (−2.0 to −1.0)	<0.0001
	6 months	60 months	0.0 (−0.9 to 1.0)	0.93
PAPi	Pre-LVAD	6 months	−0.6 (−1.1 to −0.2)	<0.01
	6 months	60 months	−0.2 (−1.0 to 0.6)	0.62

CO, cardiac output; CI, cardiac index; dPAP, diastolic pulmonary artery pressure; LVAD, left ventricular assist device; mPAP, mean pulmonary artery pressure; PAPi, pulmonary artery pulsatility index; PCWP, pulmonary capillary wedge pressure; PVR, pulmonary vascular resistance; RAP, right atrial pressure; RVSWI, right ventricular stroke work index; sPAP, systolic pulmonary artery pressure; SVR, systemic vascular resistance. *Note:* Changes represent pre-specified contrasts of estimated marginal means derived from the mixed-effects models at predefined time points (baseline vs. 6 months; 6 vs 60 months), incorporating all available longitudinal RHC measurements at their actual follow-up times. These contrasts do not represent pairwise comparisons between sequential RHC measurements.No multiplicity adjustment was applied

SVR decreased early after implantation (−292 dynes·s/cm⁵, 95% CI −422 to −161, *p*<0.0001), as did PVR (−62 dynes·s/cm⁵, 95% CI −82 to −42, *p*<0.0001); both remained stable thereafter. Cardiac output and cardiac index increased at 6 months (CO +1.3 L/min; CI +0.6 L/min/m²; both *p*<0.0001) and showed no subsequent changes.

RVSWI decreased significantly from baseline to 6 months (−1.5 g·m/m²/stroke, *p*<0.0001), with no significant change between 6 and 60 months. Similarly, PAPi decreased modestly from baseline to 6 months (−0.6, *p*<0.01), without further change thereafter.

NYHA functional class improved predominantly within the first 6 months after LVAD implantation and remained relatively stable during longer-term follow-up (Supplementary Figure 2).

### Subgroup analyses of hemodynamic trajectories

Hemodynamic trajectories stratified by survival status are shown in Figure 2. Non-survivors exhibited higher mPAP, PCWP, and RAP over time compared with survivors (interaction *p*=0.08, *p*=0.03, and *p*=0.02, respectively). PAPi trajectories also differed between survivors and non-survivors (interaction *p*=0.08), with PAPi values remaining consistently below 2 in non-survivors. CO and CI did not differ significantly between groups (interaction *p*=0.40 and *p*=0.59). Additional hemodynamic parameters (sPAP, dPAP, SVR, PVR, and RVSWI) are shown in Supplementary Figure 3, with a significant interaction observed only for dPAP (interaction *p*=0.04).

**Figure 2:**
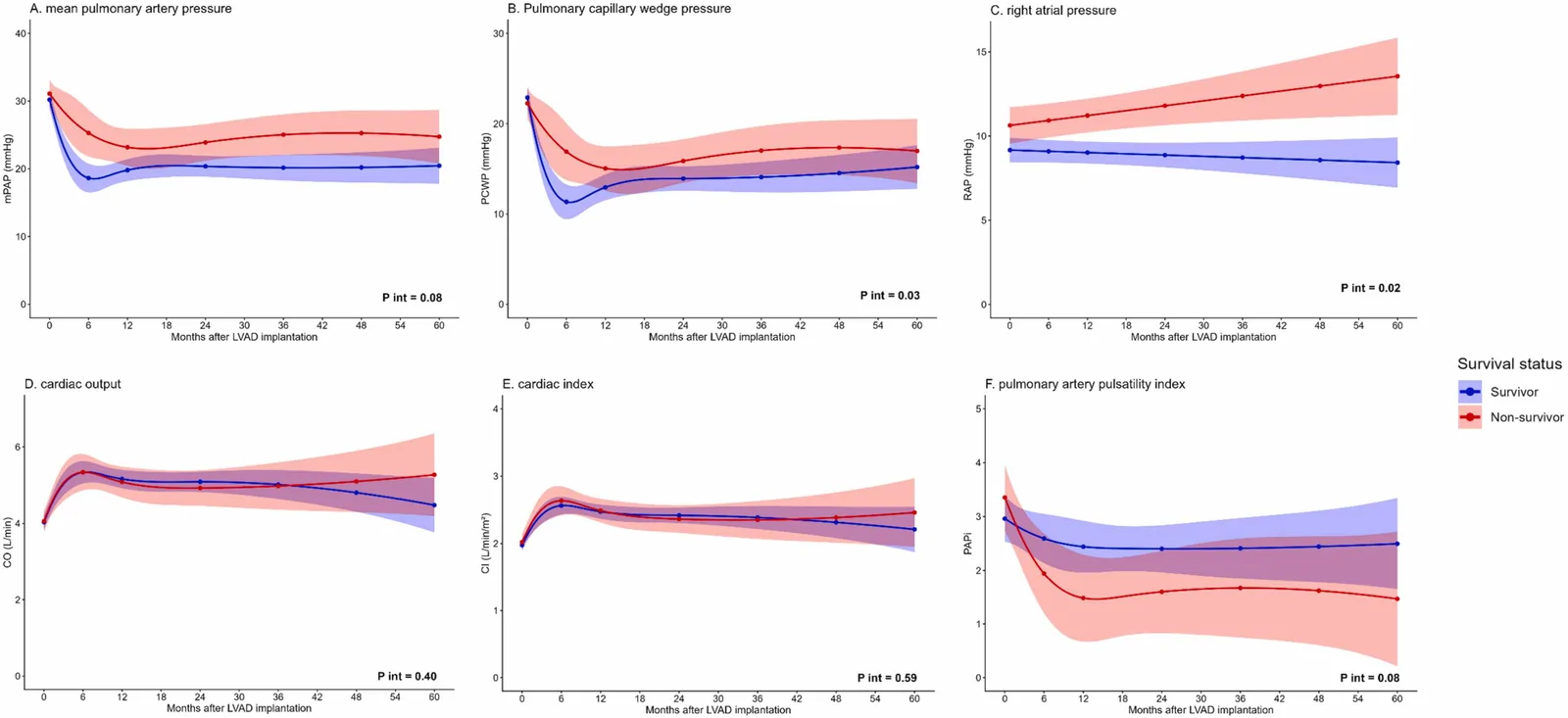
Right-sided hemodynamic trajectories by survivor status. A – mPAP; B – PCWP; C – RAP; D – CO; E – CI; F – PAPi. Trajectories were derived as described in Figure 1. CO, cardiac output; CI, cardiac index; LVAD, left ventricular assist device; mPAP, mean pulmonary artery pressure; PAPi, pulmonary artery pulsatility index; PCWP, pulmonary capillary wedge pressure; RAP, right atrial pressure

### Association between hemodynamic changes and clinical outcomes

When stratified by clinical late RVF status (Figure 3), patients with late RVF showed higher mPAP, PCWP, and RAP trajectories compared with those without late RVF (interaction *p*=0.04, *p*=0.07, and *p*=0.02, respectively). CO output and CI trajectories were similar between groups (interaction *p*=0.10 and *p*=0.24). PAPi trajectories did not differ significantly between patients with and without late RVF (interaction *p*=0.35). Corresponding additional hemodynamic parameters are presented in Supplementary Figure 4, again showing a significant interaction only for dPAP (interaction *p*<0.01).

**Figure 3:**
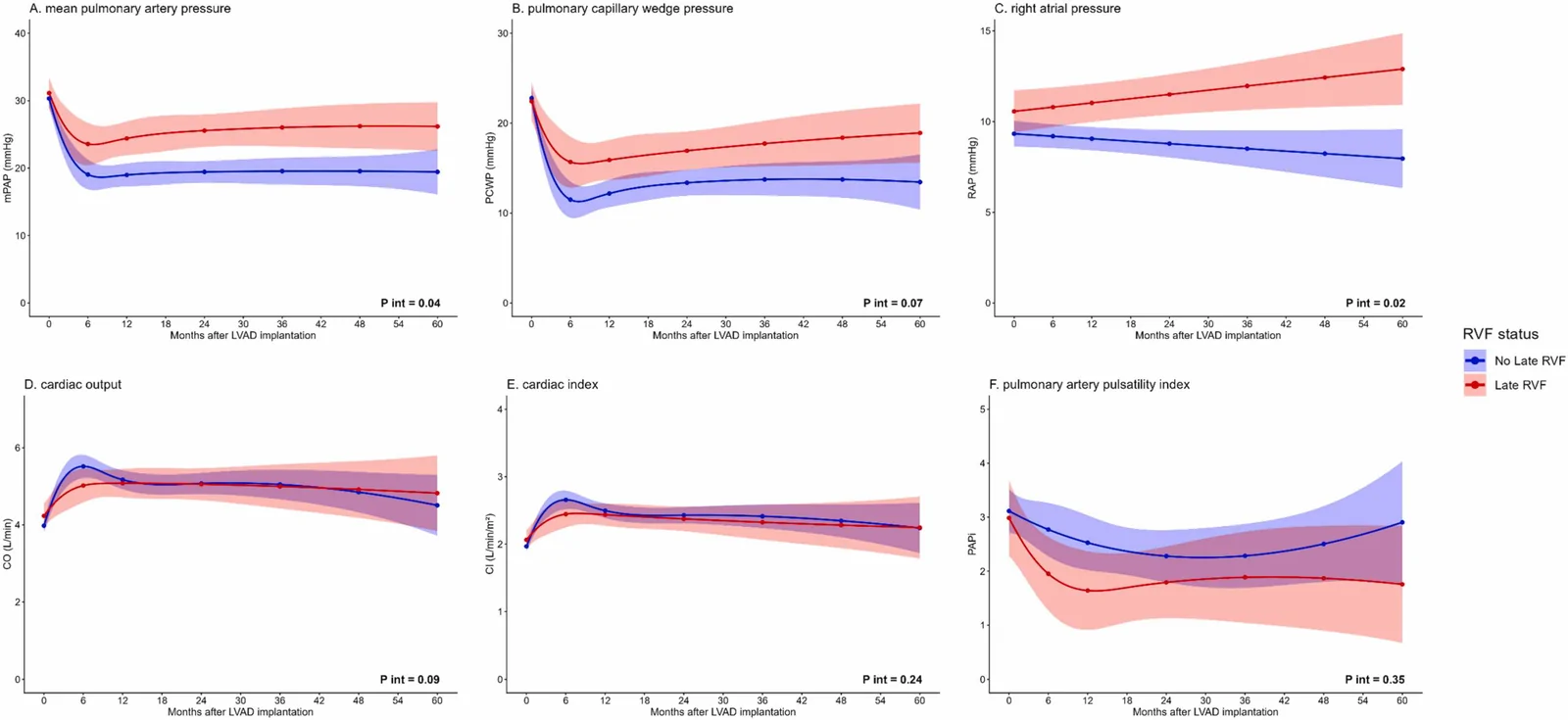
Right-sided hemodynamic trajectories by late RV Failure status. A – mPAP; B – PCWP; C – RAP; D – CO; E – CI; F – PAPi. Trajectories were derived as described in Figure 1. CO, cardiac output; CI, cardiac index; LVAD, left ventricular assist device; RV, right ventricle; mPAP, mean pulmonary artery pressure; PAPi, pulmonary artery pulsatility index; PCWP, pulmonary capillary wedge pressure; RAP, right atrial pressure

Stratification by initial LVAD strategy (BTT vs. DT/BTD) demonstrated no significant differences in hemodynamic trajectories over time (all interaction *p*≥0.10; Figure 4 and Supplementary Figure 5).

**Figure 4:**
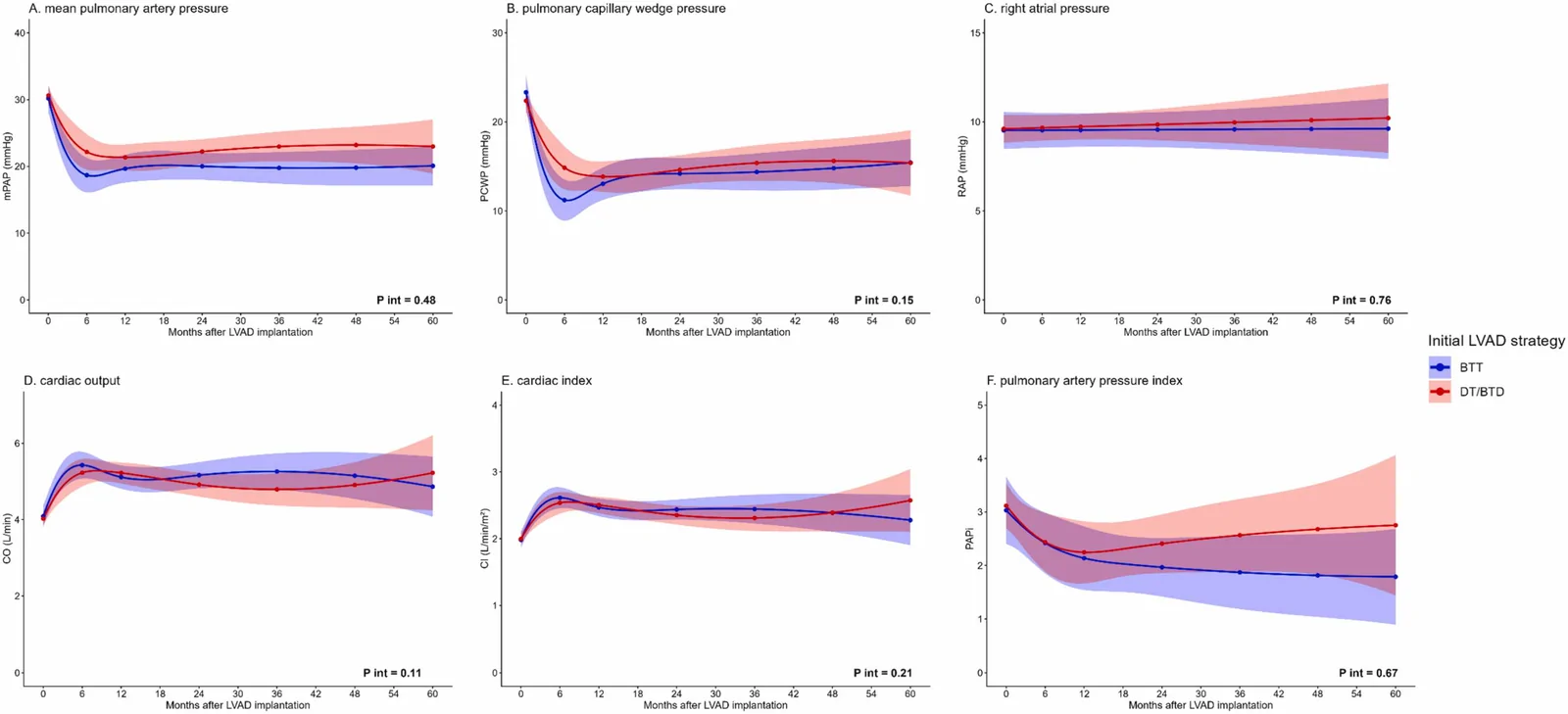
Right-sided hemodynamic trajectories by initial LVAD strategy. A – mPAP; B – PCWP; C – RAP; D – CO; E – CI; F – PAPi. Trajectories were derived as described in Figure 1. CO, cardiac output; CI, cardiac index; LVAD, left ventricular assist device; mPAP, mean pulmonary artery pressure; PAPi, pulmonary artery pulsatility index; PCWP, pulmonary capillary wedge pressure; RAP, right atrial pressure

Figure 5 and Supplementary Figure 6 show hemodynamic trajectories stratified by AR status. Significant interactions were found for RAP, sPAP, and SVR (interaction *p*=0.05, *p*=0.09, and *p*=0.08, respectively), with elevated trajectories in the clinically significant AR group. When stratified by WRF status, a significant interaction over time was observed for all pulmonary artery pressures, PCWP, RAP (Figure 6 and Supplementary Figure 7). Patients who developed WRF demonstrated persistently higher mPAP, PCWP, and RAP trajectories, compared with patients without WRF.

**Figure 5:**
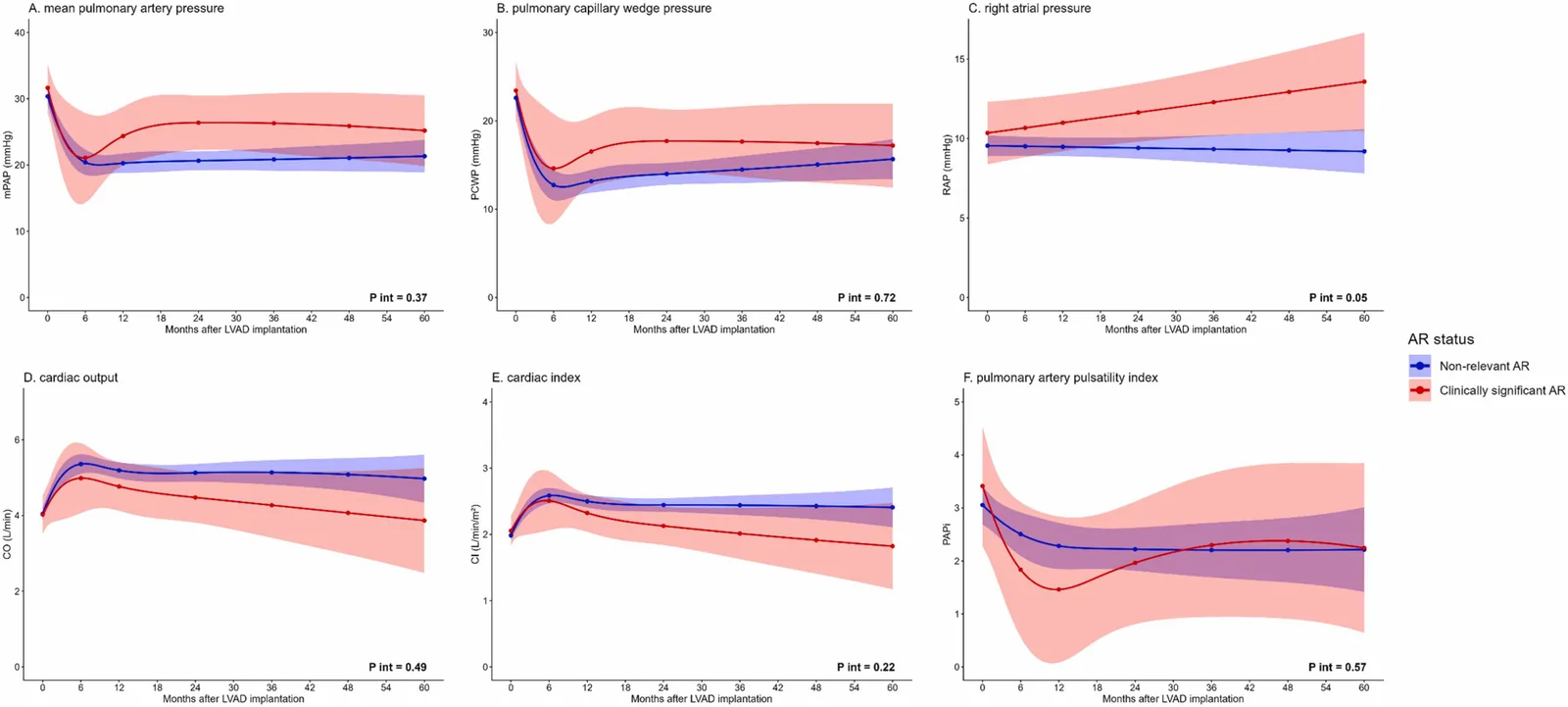
Right-sided hemodynamic trajectories according to Aortic Regurgitation status. A – mPAP; B – PCWP; C – RAP; D – CO; E – CI; F – PAPi. Trajectories were derived as described in Figure 1. CO, cardiac output; CI, cardiac index; LVAD, left ventricular assist device; AR, aortic regurgitation; mPAP, mean pulmonary artery pressure; PAPi, pulmonary artery pulsatility index; PCWP, pulmonary capillary wedge pressure; RAP, right atrial pressure

**Figure 6:**
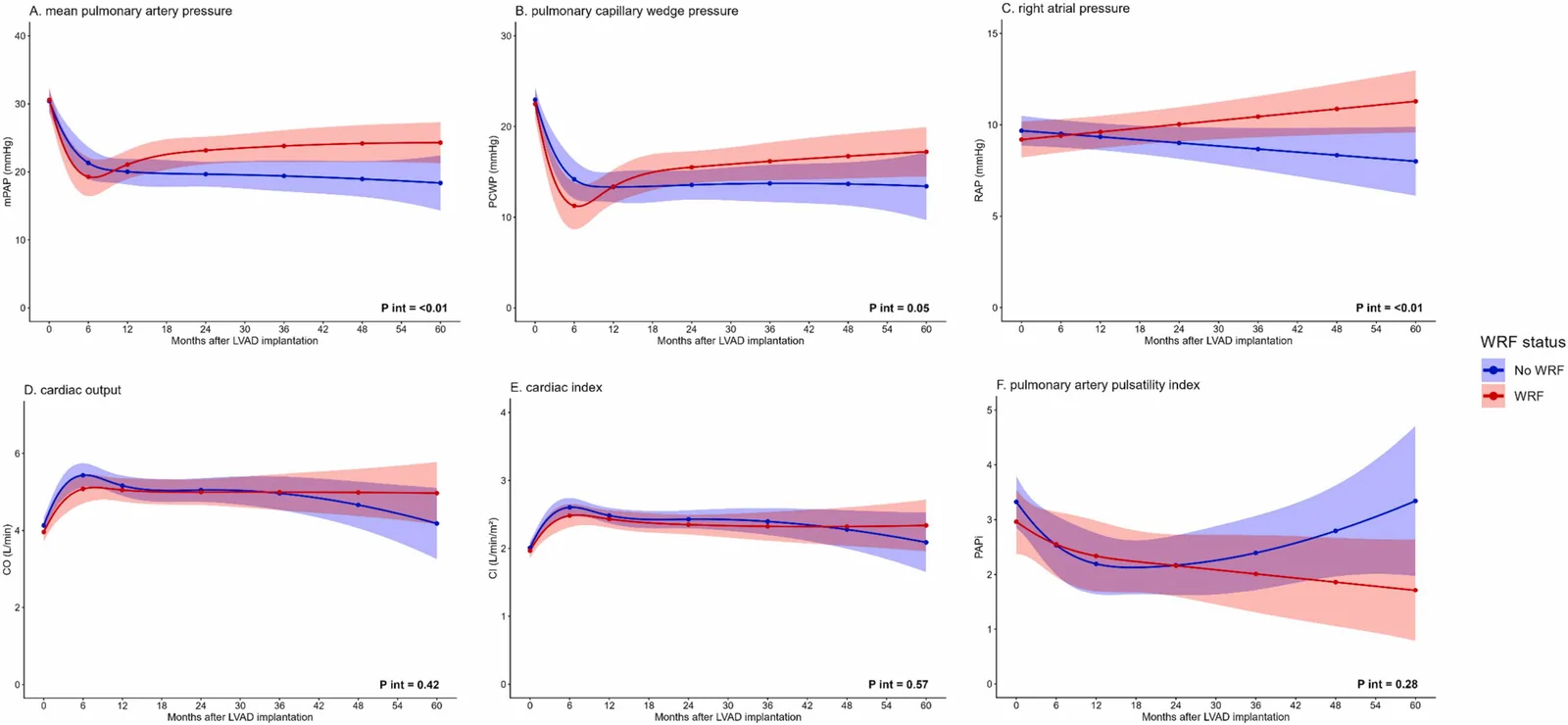
Right-sided hemodynamic trajectories according to Worsening Renal Function status. A – mPAP; B – PCWP; C – RAP; D – CO; E – CI; F – PAPi. Trajectories were derived as described in Figure 1. CO, cardiac output; CI, cardiac index; LVAD, left ventricular assist device; WRF, worsening renal function; mPAP, mean pulmonary artery pressure; PAPi, pulmonary artery pulsatility index; PCWP, pulmonary capillary wedge pressure; RAP, right atrial pressure.

## Discussion

This study describes the longitudinal trajectories of invasive hemodynamic parameters following LVAD implantation in a medium-volume center in the Netherlands. To our knowledge, this represents the first detailed longitudinal analysis of invasive hemodynamic trajectories during long-term support in a predominantly HeartMate 3 population. Previous work has described longitudinal hemodynamic changes during LVAD support, but was primarily based on earlier-generation devices and relied on descriptive longitudinal trends, supplemented by comparisons across broadly categorized follow-up intervals.^24^ The present study extends these observations with a more detailed longitudinal characterization of invasive hemodynamic trajectories. We observed early and sustained reductions in pulmonary and left-sided cardiac filling pressures, accompanied by an increase in CO and CI. RAP remained largely stable over time. In subgroup analyses, trajectories of mPAP, PCWP, and RAP differed according to survival status and the occurrence of late right ventricular failure. Distinct trajectories were also observed in patients with clinically significant AR and WRF, underscoring the complex and dynamic nature of hemodynamic changes during long-term LVAD support.

### Longitudinal hemodynamic changes after LVAD implantation

The early reduction in pulmonary artery pressures and PCWP observed after LVAD implantation reflects the primary hemodynamic effect of mechanical left ventricular unloading, consistently demonstrated in previous studies.^8^, ^25^ Our data show that this unloading effect is largely sustained over prolonged follow-up, with parallel decrease in systemic and pulmonary vascular resistance and persistent increases in CO and CI, indicating durable hemodynamic efficiency during long-term LVAD support.^8^, ^26^ However, PCWP demonstrated a modest increase between 6 and 60 months, suggesting that the initial reduction in left-sided filling pressures may attenuate over time in some patients. A similar temporal pattern was observed for functional status, with NYHA functional class improving predominantly within the first 6 months and remaining relatively stable thereafter, although the relationship between functional and hemodynamic changes was not formally assessed.

Notably, RAP remained largely stable over time despite increased forward flow and decreased left ventricular preload. This finding may in part reflect patient selection, as individuals with progressive right-sided congestion are less likely to survive to later follow-up time points.^27^, ^28^ In addition, long-term RHC follow-up was predominantly available in BTT patients undergoing routine transplant surveillance, potentially resulting in an overrepresentation of clinically stable patients at later time points. Conversely, repeat RHCs in DT patients were generally performed only when clinically indicated, which may have enriched the cohort for patients with clinical deterioration. The net effect of these opposing selection mechanisms on long-term RV trajectories therefore remains uncertain. Finally, the absence of rising RAP may indicate effective clinical management of right ventricular loading conditions during follow-up, particularly optimization of volume status.

In contrast, indices of right ventricular performance demonstrated a distinct temporal pattern, with an early reduction in RVSWI and a modest decline in PAPi from LVAD implantation to 6 months, followed by stabilization thereafter. These changes likely reflect altered right ventricular-pulmonary artery coupling in the context of reduced pulmonary vascular load and RV adaptation rather than isolated progressive RV deterioration.^28^, ^29^ Although RVSWI is incorporated into preoperative risk models for post-LVAD RV failure, its incremental value for assessing right ventricular performance after LVAD implantation is limited by markedly altered loading conditions and RV hemodynamics, and there is no consensus on its use as a marker of intrinsic RV function during long-term LVAD support.^28^, ^29^, ^30^ While some studies have reported progressive RV dysfunction during long-term LVAD support, our findings point toward relative hemodynamic stabilization in selected long-term survivors, highlighting the dynamic and heterogeneous nature of RV adaptation after LVAD implantation.^24^, ^28^, ^31^, ^32^

### Hemodynamic differences in subgroup analyses

Subgroup analyses demonstrated distinct hemodynamic trajectories according to survival status and the occurrence of late RVF. Non-survivors and patients who developed late RVF showed persistently higher mPAP, PCWP, and RAP over time compared with their respective reference groups. Patients with persistently elevated RAP or unfavorable right-left filling pressure ratios have been shown to have higher risk of death or right ventricular support post-implantation.^27^, ^28^ In both stratifications, mPAP and PCWP decreased early after LVAD implantation but remained higher in the groups with adverse clinical events, whereas RAP increased early after implantation. Given the strong association between RVF and mortality after LVAD implantation, the marked similarity between the survival-based and RVF-based hemodynamic trajectories is not unexpected.^33^, ^34^ In contrast, trajectories of CO and CI were similar across survival and RVF strata, suggesting that differences in outcomes were not driven by impaired forward flow alone.^35^ This pattern may reflect incomplete left ventricular unloading due to right ventricular constraints, resulting in relative under-unloading (pump-patient mismatch) despite preserved cardiac output.

A lower PAPi has been shown to be a strong predictor of RVF after LVAD implantation, as well as the severity of RVF.^28^ Although no definitive PAPi cut-off has been established, several studies have suggested that values below 2 are consistently associated with adverse outcomes, including increased mortality.^36^, ^37^ In the present study, PAPi values in non-survivors remained consistently below 2 following LVAD implantation, in line with these prior observations. In contrast, PAPi trajectories in patients with late RVF showed a similar pattern, although the interaction did not reach statistical significance. These findings suggest that PAPi may reflect aspects of global hemodynamic vulnerability beyond formal definitions of late RVF. Other hemodynamic parameters did not differ meaningfully across subgroups.

Clinically significant AR and WRF were associated with unfavorable hemodynamic trajectories during long-term LVAD support and are established contributors of hemodynamic deterioration and adverse outcomes in advanced HF.^38^, ^39^ Patients who developed WRF demonstrated persistently higher mPAP, PCWP, and RAP trajectories compared with patients without WRF, mirroring the hemodynamic patterns observed in non-survivors and those with late RVF. These findings support persistent congestion and adverse right-sided loading conditions as central features of hemodynamic deterioration during durable LVAD support. Hemodynamic deterioration in LVAD patients likely reflects the cumulative effect of multiple, often subtle, interacting processes, including valve pathology, RV adaptation, renal function, neurohormonal activation, and device-patient interactions.^38^, ^39^, ^40^, ^41^, ^42^

Interestingly, pre-implant hemodynamic profiles were broadly similar between patients with and without adverse clinical outcomes, whereas differences became more apparent during LVAD support. This observation highlights the potential relevance of longitudinal hemodynamic assessment beyond pre-implant measurements alone.

Given the observational design and non-protocolized timing of RHC, these findings should be interpreted as descriptive associations rather than evidence of causality or prediction.

Finally, the absence of differences in hemodynamic trajectories according to initial LVAD strategy indicates that long-term invasive hemodynamic profiles were similar between BTT and DT/BTD recipients, despite differences in baseline characteristics at implantation. This suggests that differences in clinical outcomes between LVAD strategies may be more attributable to baseline patient risk profiles rather than intrinsic differences in hemodynamic adaptation.^38^, ^43^ To our knowledge, direct strategy-based longitudinal comparisons of serial invasive hemodynamics between BTT and DT/BTD groups have not previously been described.

## Limitations

Several limitations should be acknowledged. First, longitudinal analyses are subject to survivorship bias, as later time points include fewer patients and predominantly reflect individuals with more favorable outcomes. Although mixed-effects models accommodate unbalanced longitudinal data, they cannot fully account for informative dropout due to death or transplantation. Second, RHCs were performed as part of routine clinical care rather than a fully standardized protocol. Although patients listed for heart transplantation underwent assessments at predefined intervals, repeat RHCs in destination therapy patients were performed only when clinically indicated, potentially resulting in an overrepresentation of clinically unstable patients. Furthermore, timing and frequency varied across the overall cohort, limiting direct comparability across time points. In addition, limited statistical power may have reduced the ability to detect differences in subgroup and interaction analyses. Changes in medical therapy and LVAD setting were managed according to standard practice and were not accounted for in the analyses, which may have influenced individual hemodynamic measurements. Third, RV failure was defined using established clinical criteria, but incomplete availability of certain parameters (e.g., lactate levels and mixed venous oxygen saturation) may have led to misclassification. Finally, this single-center design may limit generalizability, although the consistency of findings with prior reports supports their external validity.

## Conclusion

In this single-center longitudinal study, LVAD implantation was associated with early and sustained reductions in pulmonary and left-sided cardiac filling pressures, accompanied by stable long-term hemodynamic profiles during follow-up. Distinct hemodynamic trajectories were observed among patients who died or developed late RVF, characterized by persistently higher left- and right-sided filling pressures and lower PAPi. In addition, clinically relevant AR and WRF were each associated with distinct patterns of hemodynamic trajectories affecting different pressure and resistance domains. Collectively, these findings highlight the heterogeneity of hemodynamic adaptation during LVAD support and demonstrate that distinct longitudinal hemodynamic trajectories are associated with adverse clinical outcomes. The potential clinical utility of serial invasive hemodynamic assessment warrants prospective evaluation.

## Disclosures

SL has nothing to disclose. The employer of AAV received consultancy fees and/or research support from Adrenomed, AnaCardio, AstraZeneca, Bayer AG, BMS, Boehringer Ingelheim, Corteria, Eli Lilly, Merck, Moderna, Novartis, Novo Nordisk, Roche Diagnostics and Salubris Bio. KD reports speaker and consultancy fees to institution from Boehringer Ingelheim, AstraZeneca, Abbott, Novartis, FIRE1, and Echosense. JMtM is supported by a Veni grant from ZonMW as well as a Dekker grant from the Dutch Heart Foundation and declares consulting and/or speaker fees to institution from Astra Zeneca, Bayer, Boehringer Ingelheim, Novo Nordisk, and Roche, and served on the data safety monitoring board for FASTR. JKRVK reports speaker fees from Boehringer Ingelheim and Abbot.

## Declaration of Competing Interest

The authors declare the following financial interests/personal relationships which may be considered as potential competing interests: Kevin Damman reports a relationship with Abbott that includes: speaker and consultancy fees to his institution. If there are other authors, they declare that they have no known competing financial interests or personal relationships that could have appeared to influence the work reported in this paper.

## Footnotes

Appendix A Supplementary data associated with this article can be found in the online version at doi:10.1016/j.jhlto.2026.100711.
