# Supplementary material for: Long-term trajectories of invasive hemodynamic parameters and clinical outcomes after durable left ventricular assist device implantation
Source: JHLT Open. 2026 Oct 21;15:100711. doi: 10.1016/j.jhlto.2026.100711 (PMC13753594; doi:10.1016/j.jhlto.2026.100711)
Supplement: Supplementary file 1 [file mmc1.docx]

**Supplementary material**

**Supplementary Table 1. Number of Events**

| **Outcome** | **N (%)** | **Median time to event (months)** |
| --- | --- | --- |
| Death * | 66 (33) | 21 [4 – 42] |
| Late RVF | 48 (24) | 17 [9 - 26] |
| Clinically relevant AR | 19 (10) | 12 [6-24] |
| Worsening renal function | 74 (37) | 6 [6-12] |
| * 15 deaths (23%) were directly attributed to right-heart failure. When including deaths classified as cardiopulmonary failure, 23 deaths (35%) were considered potentially related to right ventricular dysfunction | | |

**Supplementary Figure 1. Number of Right Heart Catheterizations per timepoint by initial VAD strategy**

**Supplementary Figure 2. NYHA functional class during longitudinal follow-up after LVAD implantation**

*Distribution of New York Heart Association (NYHA) functional class before LVAD implantation and at 6, 12, 24, 36, 48, and 60 months after implantation. Percentages are calculated among patients with an available NYHA assessment at each time point. The number of patients with available NYHA class is shown below each time point.*

**Supplementary Figure 3. Additional right-sided hemodynamic trajectories according to survivor status**

**Supplementary Figure 4. Additional right-sided hemodynamic trajectories according to late RV failure status**

**Supplementary Figure 5. Additional right-sided hemodynamic trajectories according to initial LVAD strategy**

**Supplementary Figure 6. Additional right-sided hemodynamic trajectories according to Aortic Regurgitation status**

**Supplementary Figure 7. Additional right-sided hemodynamic trajectories according to Worsening Renal Function status**
